# ADDRESSING ALZHEIMER’S DISPARITIES AMONG BLACK POPULATIONS WITH BRAINGUIDE BY USAGAINSTALZHEIMER’S

**DOI:** 10.1093/geroni/igac059.1866

**Published:** 2022-12-20

**Authors:** Brooks Kenny

**Affiliations:** UsAgainstAlzheimer's, Kensington, Maryland, United States

## Abstract

Older, Black Americans are disproportionately impacted by Alzheimer’s disease (AD), accounting for 15% (~1 million) of all individuals aged 65+ living with AD (5.8 million). Stigma, fear, and gaps in education contribute to 60% of undetected AD cases. In Georgia, AD remains the 6th leading cause of death. By 2029, cases are projected to spike by 46%, from 130,000 to 190,000. Given these alarming statistics and in response to AD health disparities in this population, UsAgainstAlzheimer’s, in partnership with community leaders and organizations, launched a pilot outreach program to promote AD prevention and brain health awareness in Atlanta. Program goals included: increasing knowledge about brain health, emphasizing the importance of early detection and diagnosis, raise awareness of BrainGuide™ by UsAgainstAlzheimer’s and other brain health resources, and develop a network of organizations for ongoing collaboration, awareness, and education. Program strategies included accessing highly saturated, faith-based spaces like mega churches, circulating key messaging through paid and earned media, and hosting widely received community webinars. UsAgainstAlzheimer’s collected participant feedback and examined BrainGuide website traffic to evaluate the effectiveness of community engagement on increasing brain health awareness and addressing AD stigma in Atlanta. Preliminary findings indicate a 96% increase in BrainGuide traffic from Atlanta and 70% increased engagement with BrainGuide resources, compared to the national average. UsAgainstAlzheimer’s’ pilot program suggests that brain health promotion, grounded in community engagement from trusted influencers, has potential to raise brain health awareness and empower people to take action. Further research and learnings are required to determine program scalability.

